# Recent Patents of Interest to Dentists

**Published:** 1906-10

**Authors:** 


### RECENT PATENTS OF INTEREST TO DENTISTS.

829275, Tooth-powder can, John E. Lee, Conshohocken, Pa.
829119, Dental plugger, Frank X. Melen, Triadelphia, Ohio.
829395, Dental engine, Nathan K. Carhart, Indianapolis, Indiana.
829449, Tooth-brush, Wm. L. Whaley and H. L. Loring, Chicago, Ill.
829997, Manufacture of plates for artificial teeth, Arthur Ollendorff, Breslau, Germany.
830872, Amalgam carrier and plugger, Hugh W. Arthur, Pittsburg, Pa.
820852, Mechanism for cutting wooden toothpicks, John M. Rounds, Strong, Maine.
831185, Vitreous cement for plugging teeth and manufacturing the same, Josef Rawitzer, Charlottenburg, Ger.
831307, Dental tool, Charles A. Spahn, Newark, N. J.
831489, Dental disk holder, Jacob A. Thomas, Hanover, Pa.
831391, Attachment for tooth-brushes, &c., James L. Ubellar, Kankakee, Ill.

Copies of above patents may be obtained for ten cents each, by addressing John A. Saul, Solicitor of Patents, Fendall Building, Washington, D. C.

